# Innovative method for encapsulating highly pigmented biomass from *Aspergillus nidulans* mutant for copper ions removal and recovery

**DOI:** 10.1371/journal.pone.0259315

**Published:** 2021-11-02

**Authors:** Ailton Guilherme Rissoni Toledo, Jazmina Carolina Reyes Andrade, Mauricio Cesar Palmieri, Denise Bevilaqua, Sandra Regina Pombeiro Sponchiado

**Affiliations:** 1 Department of Biochemistry and Organic Chemistry, Institute of Chemistry, São Paulo State University-UNESP, Araraquara, SP, Brazil; 2 Itatijuca Biotech, São Paulo, SP, Brazil; King Saud University, SAUDI ARABIA

## Abstract

Biosorption has been considered a promising technology for the treatment of industrial effluents containing heavy metals. However, the development of a cost-effective technique for biomass immobilization is essential for successful application of biosorption in industrial processes. In this study, a new method of reversible encapsulation of the highly pigmented biomass from *Aspergillus nidulans* mutant using semipermeable cellulose membrane was developed and the efficiency of the encapsulated biosorbent in the removal and recovery of copper ions was evaluated. Data analysis showed that the pseudo-second-order model better described copper adsorption by encapsulated biosorbent and a good correlation (r^2^ > 0.96) to the Langmuir isotherm was obtained. The maximum biosorption capacities for the encapsulated biosorbents were higher (333.5 and 116.1 mg g^-1^ for EB10 and EB30, respectively) than that for free biomass (92.0 mg g^-1^). SEM-EDXS and FT-IR analysis revealed that several functional groups on fungal biomass were involved in copper adsorption through ion-exchange mechanism. Sorption/desorption experiments showed that the metal recovery efficiency by encapsulated biosorbent remained constant at approximately 70% during five biosorption/desorption cycles. Therefore, this study demonstrated that the new encapsulation method of the fungal biomass using a semipermeable cellulose membrane is efficient for heavy metal ion removal and recovery from aqueous solutions in multiple adsorption-desorption cycles. In addition, this reversible encapsulation method has great potential for application in the treatment of heavy metal contaminated industrial effluents due to its low cost, the possibility of recovering adsorbed ions and the reuse of biosorbent in consecutive biosorption/desorption cycles with high efficiency of metal removal and recovery.

## Introduction

Various anthropogenic and industrial activities generate bulk quantities of waste containing considerable concentrations of heavy metals, which have detrimental effects on terrestrial and aquatic environments for all living beings [1, 2]. Copper ion is one of the most common heavy metals in effluents from different industries and it can become toxic to cells when its concentrations surpass certain optimal levels, causing adverse human health effects [3, 4]. After introducing more restrictive laws for wastewater disposal contaminated with metals, economic, effective and eco-friendly technology needed to be developed to remove toxic metals from wastewater before disposing of it safely.

Compared to conventional methods (precipitation, flocculation, ion exchange and membrane filtration), biosorption has been considered a promising alternative to treat large amounts of industrial effluents containing heavy metals in low concentrations [3, 5, 6]. The main advantages for biosorption applications in industrial processes are the low cost of biosorbents, their high efficiency for metal removal (especially in low-concentration solutions), regeneration/reuse of biosorbents, potential metal recovery, and the non-generation of secondary residues [7–12].

Among the various types of biosorbents, fungal biomass has been considered as a cost-effective adsorbent for treating metal-contaminated wastewaters because it can be easily obtained in large quantities from industrial processes or organisms of rapid growth using simple and inexpensive cultivation techniques [2, 7, 13–16]. Several studies have reported fungal biomass as a promising biosorbent for heavy metal removal from industrial wastewater [9, 11, 13, 17–21].

As the biosorption consists of the adsorption of metals into the cellular surface of the biomass, the metal binding capacity depends mainly on the components present on the cell surface and the spatial structure of the cell wall [5, 6, 22, 23]. The fungal cell walls are complex macromolecular structures predominantly consisting of chitin, glycans, mannans, which have various functional groups (amine, imidazole, phosphate, sulfate, sulfhydryl and hydroxyl) that are potential metal-binding sites. Furthermore, some fungal species produce a dark-brown pigment closely associated with chitin, known as melanin that contains many groups including carboxyl, phenolic and alcoholic hydroxyl, carbonyl and methoxyl, which have a vital role in metal adsorption, significantly increasing the efficiency of the biosorption process [18, 24–27].

Studies in our laboratory showed that the highly pigmented biomass produced by the MEL1 mutant from *Aspergillus nidulans* has a higher biosorption capacity for neodymium than the unpigmented biomass [28]. We characterize this pigment as 3,4-dihydroxyphenylalanine (DOPA)-melanin according to its physicochemical properties and tests with melanin biosynthesis inhibitors [29]. In the literature, other studies have also suggested that biomass from fungi pigmented can be considered a promising biosorbent due to the fact that melanin acts as a metal chelator, significantly enhancing the biomass-metal interaction and consequently its biosorption capacity [18, 30–34].

For the application of fungi biomass in large-scale processes, immobilization of biosorbent is a necessary step to increase the efficiency of metal adsorption on the surface of biomass, the removal and recovery of metals, as well as to regenerate and reuse the biosorbent in subsequent cycles. The free microbial cells are generally small particles that have low density, poor mechanical strength and little rigidity, which may cause solid–liquid separation problems, possible biomass swelling, inability to regenerate/reuse, clogging of filter parts and a reduction in high pressure required to generate suitable flow rates in a packed or fluidized column mode [5, 35, 36]. To address these issues, microbial biomass immobilization systems, including entrapment, adsorption, cross-linking, covalent bonding to the carrier and encapsulation, have been studied [5, 37–46]. In most studies, biomass is immobilized in polymeric matrices, such as sodium alginate, polysulfone, polyacrylamide and polyurethane, with an appropriate mechanical strength porosity and size [40]. Nevertheless, this type of immobilization has some disadvantages, including mass transfer limitations and high cost of these matrices, which may not allow its application in large scale processes. Furthermore, these matrices may reduce the removal capacity, obstructing or damaging the metal-binding sites due to irreversible binding between the biosorbent and the immobilizing matrix [40, 47].

In this context, the development of a cost-effective immobilization technique for metal removal/recovery is essential to improve the competitiveness of industrial processes, decreasing the process cost and dependence on a continuous supply of biosorbent [2, 11, 40, 47]. In the present study, a new method of reversible encapsulation was developed using cheap, nontoxic and readily available semipermeable membrane capsules, in which the fungal biomass can freely float inside the capsule filled with deionized water, without blocking its binding sites and also allowing the passage of copper ions from the solution into the capsule containing the biosorvent immersed in the aqueous phase. Batch biosorption experiments were conducted to evaluate copper ion removal by an encapsulated biosorbent. The biosorption mechanism was investigated using kinetic and isothermal models, as well as scanning electron microscopy with an energy dispersive X-ray analytical system (SEM-EDXS) and FT-IR spectroscopy. The potential of our encapsulated biosorbent for a practical application in wastewater treatment was evaluated in relation to the efficiency of metal recovery in several biosorption/desorption cycles.

## Materials and methods

### Fungal cultivation

The biomass obtained after the growth of the MEL1 mutant of the fungus *Aspergillus nidulans*, characterized as an overproducer of the DOPA-melanin pigment [29], was used as a biosorvent in this study. This microorganism belongs to the culture collection of the Filamentous Fungi Laboratory at the Department of Biochemistry and Organic Chemistry, the Institute of Chemistry, São Paulo State University-UNESP in Araraquara, Brazil. Cultivation of the MEL 1 mutant was conducted as described by Sponchiado et al., 2018 [48]. After the growth period, the biomass was harvested by filtration, washed with deionized water, dried at 55°C until constant weight, crushed and sieved to obtain an adsorbent with a uniform particle size. The fraction with a diameter less than 0.42 mm was selected to be used in the sorption experiments.

### Biosorbent encapsulation

**Fig 1:**
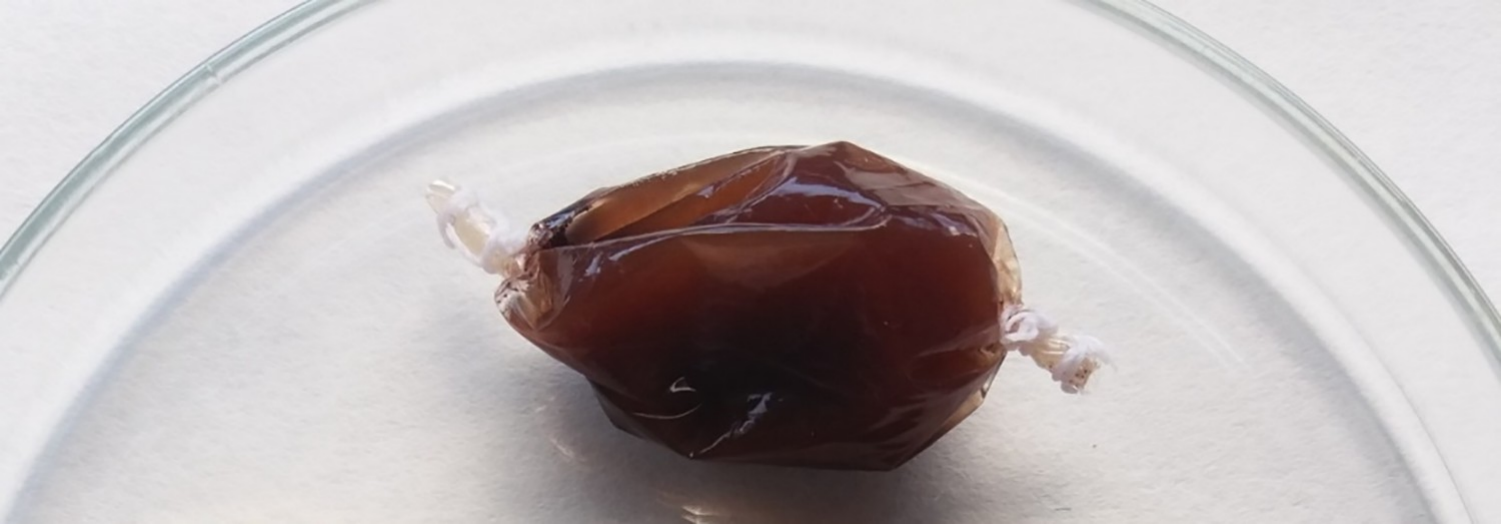
Semipermeable cellulose membrane capsule containing 30 mg of biosorbent per mL of deionized water (EB30).

The fungal biomass was enclosed in a cellulose semipermeable membrane measuring 21 mm wide and 33 mm in length containing deionized water and the system was sealed with a nylon line, forming the encapsulated biosorbent (Fig 1).

The capsules were prepared using two biosorbent concentrations. The capsule referred to as “EB10” contained 33.3 mg of biosorbent and one denominated as “EB30” contained 100 mg of biosorbent, both containing 3.33 mL of deionized water inside the capsule, whose final concentrations were 10 mg mL^-1^ and 30 mg mL^-1^, respectively.

### Biosorption studies

Biosorption kinetic experiments were performed in capped plastic bottles containing one capsule (EB10 or EB30) and 300 mL of aqueous copper solution at an initial concentration of about 100 mg L^-1^ with pH adjusted to 5.0 ± 0.1. The bottles were incubated on a rotary shaker under constant agitation of 150 rpm at room temperature (25 ± 2°C). Afterwards, a bottle was removed at each different time of incubation and the copper ion concentration in the solution was determined by Atomic Absorption Spectroscopy (Agilent Technologies 200 Series AA). Control biosorption assays were performed using a capsule containing only water (without the biosorbent) to evaluate a possible adsorption of metals by the cellulose membrane.

Isothermal studies using free and encapsulated biosorbents (EB10 or EB30) were conducted with a copper solution at different initial concentrations until the time required for the system to reach equilibrium, as determined by biosorption kinetics. During this period, the pH of this solution remained relatively constant at 5.0 ± 0.1 by adding small amounts of NaOH_(aq)_. After the equilibrium time, the remaining copper concentration was measured by the method below.

The biosorption capacity and the removal efficiency of metal ions were calculated according to Eq 1 and 2:

$$q(mg.g^{-1})=\frac{(C_{0}-C_{f}).V}{m}$$ (1)

$$Removal\;efficiency\;(\%)=\frac{C_{0}-C_{f}}{C_{0}}100$$ (2)

where q (mg g^-1^) is the biosorption capacity, m (g) is the mass of biosorbent, V (L) is the volume of the copper solution, C_0_ (mg L^-1^) is the initial copper concentration in the solution and C_f_ (mg L^-1^) is the copper concentration in the solution at the time of sampling.

From the experimental data, the pseudo-first-order and pseudo-second-order kinetic models were applied using their respective linear mathematical expressions [2, 49, 50].

$$ln\;(q_{eq}-q_{t})=ln\;q_{eq}-k_{1}t$$ (3)

$$\frac{t}{q_{t}}=\frac{t}{q_{eq}}+\frac{1}{k_{2}q_{eq}^{2}}$$ (4)

where k_1_ (min^-1^) and k_2_ (g mg^-1^ min^-1^) are the kinetic constants of pseudo first and pseudo second order of adsorption, respectively, q_eq_ and q_t_ (mg g^-1^) represent the amounts of solute adsorbed at equilibrium and time t (min), respectively.

The adsorption properties of encapsulated biosorbents at an equilibrium condition were studied by the Langmuir and Freundlich isothermal models. The Langmuir model assumes a homogeneous monolayer adsorption surface, in which the adsorption energy remains constant and the maximum adsorption capacity occurs when only a saturated layer of solute is present on the adsorbent surface [51]. The Freundlich model is widely used to describe heterogeneous multilayer adsorption surfaces with different interaction energies leading to a logarithmic decrease in affinity during surface coverage [52].

The linearized mathematical expressions of the Langmuir and Freundlich isotherms are represented in Eqs 5 and 6, respectively, as shown below:

$$\frac{C_{eq}}{q_{eq}}=\frac{1}{q_{max}K_{L}}+\frac{C_{eq}}{q_{max}}$$ (5)

$$ln\;q_{eq}=ln\;K_{F}+\frac{1}{n}ln\;C_{eq}$$ (6)

where C_eq_ (mg L^-1^) is the metal concentration at equilibrium, q_eq_ (mg g^-1^) is the biosorption capacity, amount of metal adsorbed by the biosorbents at equilibrium, q_max_ (mg g^-1^) is the maximum biosorption capacity, K_L_ (L mg^-1^) is the Langmuir constant related to the adsorption energy, K_F_ (mg^1-1/n^ L^1/n^ g^-1^) is the Freundlich constant related to the adsorption capacity and 1/n is the Freundlich constant related to the heterogeneity of the surface.

### SEM-EDXS and FTIR analysis

The biosorbent morphology before and after copper ion sorption was analysed by scanning electron microscopy (SEM, JEOL, JSM-7500F, Japan) using secondary electrons and elementary analysis of these samples was performed by dispersive X-ray spectroscopy (EDXS). Before these analyses, biosorbent samples were washed with distilled water dried at 55°C for 24 h and then coated with carbon using a vacuum system.

The functional groups present in the fungal biomass were investigated by Fourier-transform infrared spectroscopy (FT-IR). FT-IR spectra of metal-free and copper-loaded biosorbents were obtained using a Nicolet iS5 FTIR Spectrometer (Thermo Scientific). The washed and dried biomasses were mixed with KBr, pressed in a pastillator (6 tons) under vacuum for 1 min and analysed with a resolution of 2 cm^-1^ in the range of 4000–400 cm^-1^.

### Desorption studies

After the biosorption assay, the encapsulated biosorbent (EB30) was collected from the copper solution (initial concentration of 750 mg L^-1^ and pH 5) and the capsule containing the metal-loaded biomass was washed with distilled water and treated with HCl solutions at different concentrations (0.05, 0.1 and 0.2 mol L^-1^). This mixture was allowed to stand at room temperature under constant agitation (150 rpm) for 30 to 360 min to determine the time required to reach chemical equilibrium. The copper ion concentration was determined by Atomic Absorption Spectroscopy.

To evaluate the biosorption/desorption cycles, the capsules containing the metal-free biosorbent were exposed to copper solution (initial concentration of 750 mg L^-1^). After the time required to reach biosorption equilibrium, the capsules were removed, washed with distilled water and treated with HCl solution (0.05 mol L^-1^) for metal desorption. This sorption-desorption cycle was repeated five times to determine the reusability potential of the biosorbent. From the second biosorption cycle, the pH of the copper solution containing regenerated encapsulated biosorbent was corrected again at 5 using the 0.2 mol L^-1^ NaOH solution and the system was allowed to reach equilibrium once more. The cycles were performed using the same batch of capsules. The desorption capacity and recovery efficiency of the metal ions were calculated according to the following Eqs 7 and 8, respectively:

$$q_{des}=\frac{C_{f}V}{m}$$ (7)

$$d=\frac{m_{des}}{m_{bios}}100$$ (8)

where q_des_ (mg g^-1^) is the desorption capacity expressed in milligrams of metal ions desorbed per gram of biosorbent, C_f_ (mg L^-1^) is the final copper ion concentration in solution, V (L) is the volume of the solution, m (g) is the mass of the biosorbent, m_des_ (mg) is the mass of desorbed metal ions, m_bios_ (mg) is the mass of biosorbed metal ions and d (%) is the recovery efficiency expressed as a percentage of recovered metal. The m_bios_ value was obtained from the biosorption capacity calculation.

### Statistical analysis

The results were presented as the mean ± standard deviation (three independent experiments, n = 3). Root-mean-square deviation (RMSD) and linear regression analysis were used as a measure of the goodness-of-fit of the mathematical models. Small RMSE values and values of R^2^ close to 1.0 indicate better curve fitting. All kinetic and isotherm parameters of the models were evaluated by linear regression analysis of the experimental data using the Microsoft Excel 2016, version 2102, software (S1 Appendix).

## Results and discussion

For the application of biosorption in the removal of heavy metals from industrial wastewater, it is important to use immobilized biosorbent to facilitate the collection of the metal-loaded biosorbent for metal ion removal and recovery by desorption, as well as to regenerate and reuse the metal-free biosorbent for the next biosorption-desorption cycle.

In this work, the capacity of the MEL1 mutant pigmented biomass from *Aspergillus nidulans* encapsulated in a semipermeable cellulose membrane (as shown in Fig 1) for copper ion removal in aqueous solution was evaluated.

### Biosorption kinetics

**Fig 2:**
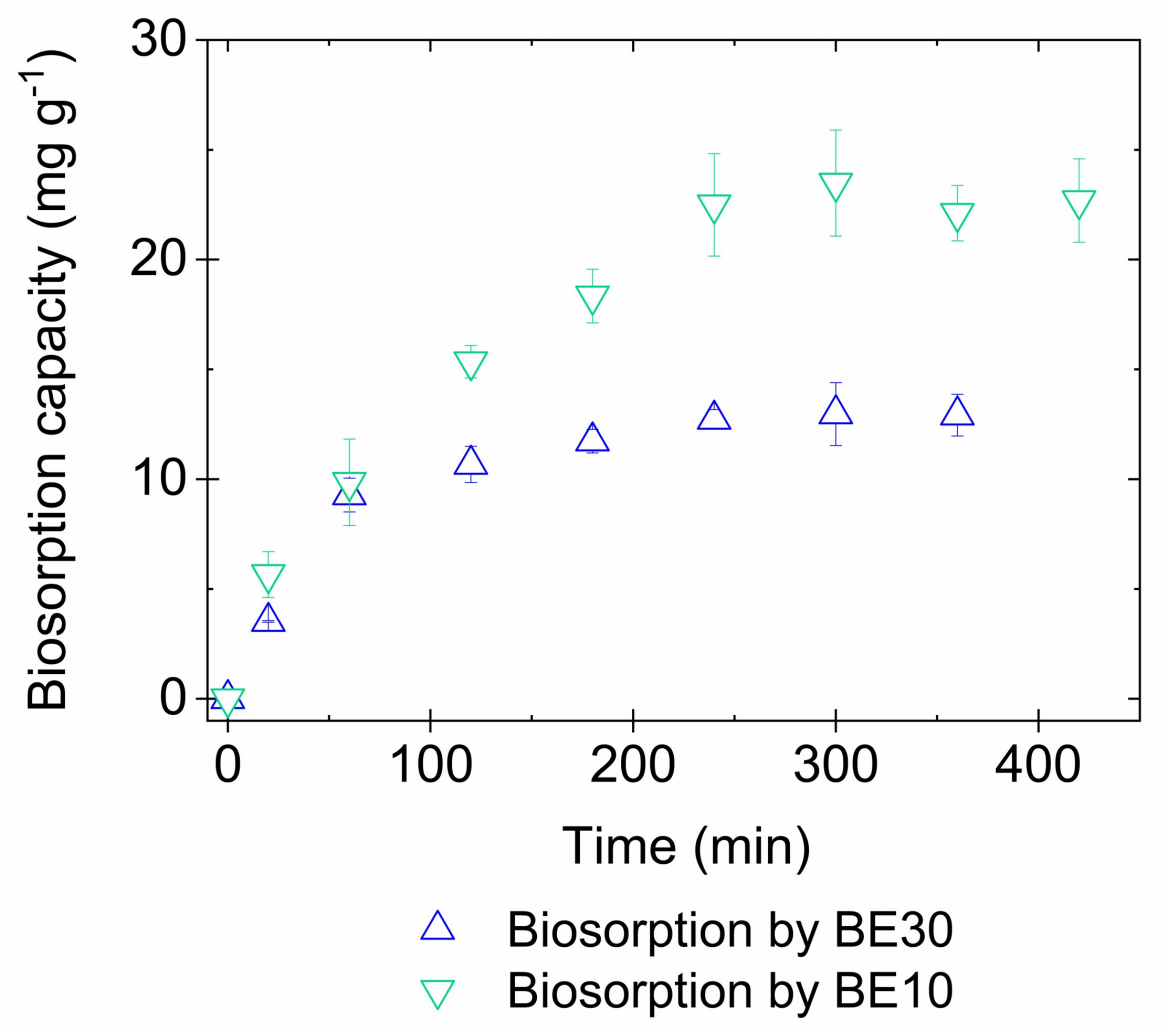
Kinetic of copper biosorption by EB30 and EB10. Conditions: Initial copper concentration = 100 mg L^-1^; pH_i_ = 5 ± 0.1; agitation speed = 150 rpm; temperature = 25 ± 2°C; biosorbent dosage for EB10 = 0.333 g L^-1^ and for EB30 = 1.0 g L^-1^.

The contact time between the biosorbent and metal ion has a considerable effect on the efficiency of the process and kinetics studies are critical to predict the adsorbent behavior for successful practical applications [2, 53]. To establish the biosorption equilibrium time, the copper sorption capacities by EB30 and EB10 encapsulated biosorbents were evaluated as a function of time. As Fig 2 shows, the amount of copper ions adsorbed increased proportional to metal–biosorbent contact time and the system reached equilibrium at 240 min. Generally, the biosorption is considered a multi-step process, comprising four consecutive elementary steps: 1- the solute transfer from the bulk solution to the liquid film surrounding the biosorbent, 2- the solute transport from the boundary liquid film to biosorbent surface (external diffusion), 3- solute transfer from the surface to the internal active binding sites (intraparticle diffusion), and 4- solute interaction with the active binding sites [40, 54].

Two kinetic models were applied to the experimental data to investigate the copper ion adsorption mechanism (data in S1 Table), which are important to select the optimum operating conditions for industrial-scale processes. The pseudo-first-order model assumes that the adsorption process is a rapid initial phase [55], while the pseudo-second-order model assumes that the adsorption is a chemical rate-controlling step [40, 50, 56].

The kinetic parameters from the linearized pseudo-first and pseudo-second order models are summarized in Table 1.

**Table 1:** Kinetic parameters for copper biosorption by EB30 and EB10. Conditions: Initial copper concentration = 100 mg L^-1^; pH_i_ = 5 ± 0.1; agitation speed = 150 rpm; temperature = 25 ± 2°C; biosorbent dosage for EB10 = 0.333 g L^-1^ and for EB30 = 1.0 g L^-1^.

Kinetic Model	Parameters	EB30	EB10
Pseudo-first order	q_eq_ (mg g^-1^)	13.2	34.4
	k_1_ (min^-1^)	0.0168	0.0169
	r^2^	0.948	0.837
	RMSD	0.592	5.12
Pseudo-second order	q_eq_ (mg g^-1^)	15.0	28.6
	k_2_ (g mg^-1^ min^-1^)	0.0013	0.0004
	r^2^	0.994	0.981
	RMSD	0.531	1.13

According to Table 1, both encapsulated biosorbents showed the highest r^2^ and the lowest RMSD values for the pseudo-second order kinetics compared to the pseudo-first order kinetic. Therefore, the pseudo-second order kinetic model best describes the experimental data, indicating that the rate limiting step in the adsorption of metals is chemisorption. Considering that an extended equilibrium time was required by encapsulated biosorbents (240 min) compared to the free biosorbent (60 min), the main rate-limiting step in copper biosorption by EB30 and EB10 is the diffusion through the capsule membrane due to the concentration gradient followed most likely by a chemical process involving valence forces between sorbent and sorbate within the capsule, such as complexation, coordination and chelation [50]. The pseudo-second order model is considered more appropriate to represent the kinetic data in several biosorption systems using fungal biomass, indicating that the rate limiting step in the adsorption of metals is chemisorption [43, 50, 57–60].

### Biosorption isotherms

**Fig 3:**
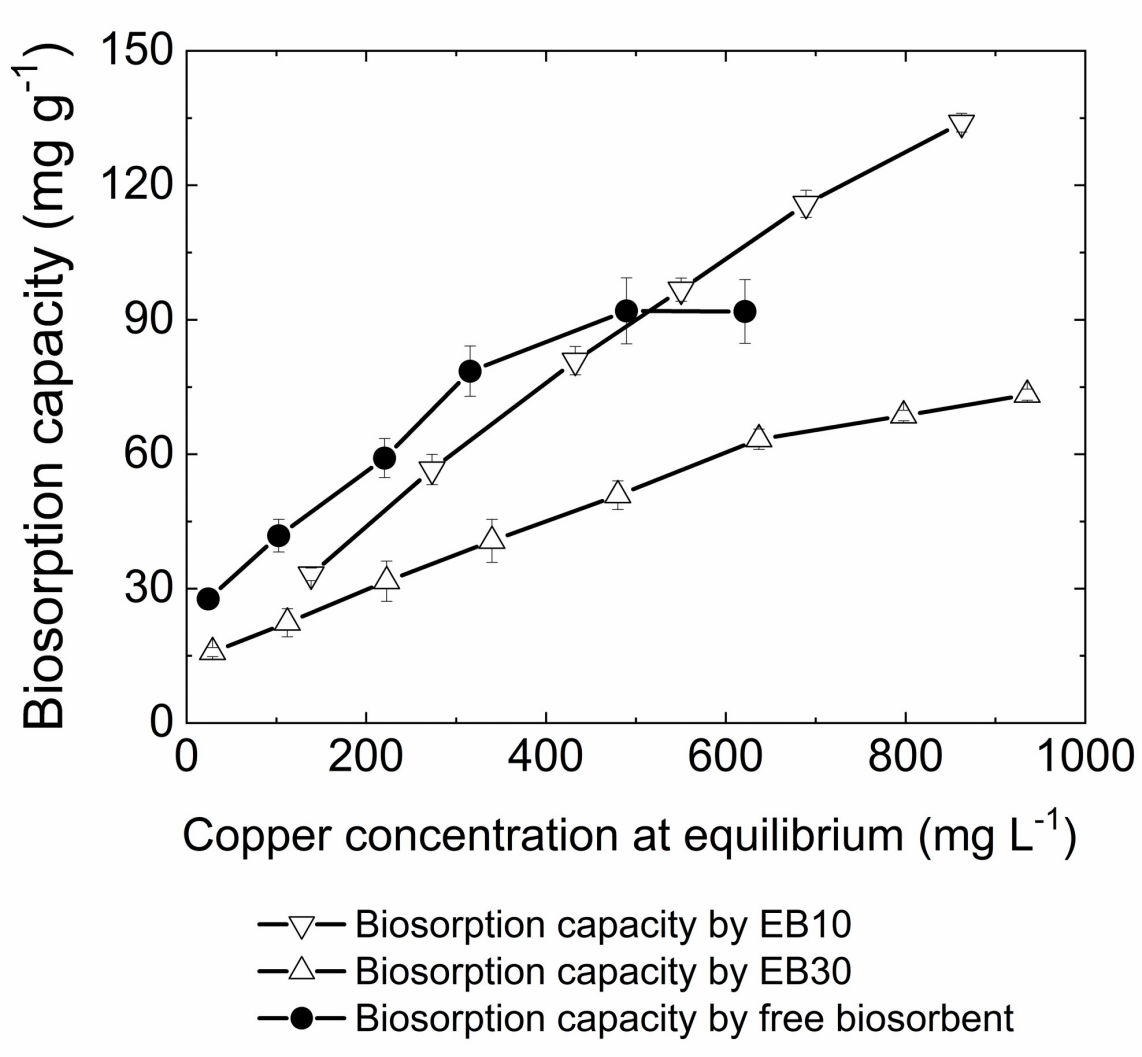
Effect of copper concentrations on the biosorption capacity by free biomass, EB30 and EB10. Conditions: Initial copper concentrations ranging from about 50 to 1050 mg L^-1^; contact time = 240 min; pH = 5.0; agitation speed = 150 rpm; temperature = 25 ± 2°C; biosorbent dosage for EB10 = 0.333 g L^-1^, for EB30 = 1.0 g L^-1^ and for free biomass = 1.0 g L^-1^.

The adsorption isotherm is important to understand how adsorbate interacts with the adsorbent and to estimate the maximum metal biosorption capacity. Fig 3 shows the experimental results regarding the effect of different copper ion concentrations on the biosorption capacity by free biosorbent, EB10 and EB30 at equilibrium.

As Fig 3 shows, in lower metal concentrations, the free biosorbent biosorptive capacity of the free biosorbent is slightly higher than the encapsulated biosorbents, probably due to the greater availability of metal to binding sites on the surface of the free biomass. The number of metal ions that bind to the biomass was lower than those that bind when there is a higher copper concentration. This phenomenon can be attributed to the thermodynamic driving force, which is proportional to the ion concentration gradient. This force is required to overcome the resistance to mass transfer of copper ions to the solid phase. Thus, in high metal ion concentrations, a higher concentration gradient increases the thermodynamic driving force, resulting in a greater probability of collision between the metal ions and the biosorbent binding sites, increasing the biosorption capacity [14, 61].

Interestingly, it can also be seen in Fig 3 that the biosorption capacity of both encapsulated biosorbents continued to increase at high copper concentrations, unlike the free biosorbent. As reported in several studies, higher concentrations of metal can lead to a greater adsorption capacity, but to a certain extent, where even with increasing metal concentration the biosorption capacity no longer increases, reaching a plateau, which indicates that the available biosorbent-binding sites are saturated [42, 60, 62–67].

The biosorption capacity by encapsulated biosorbents did not reach a plateau even as the copper concentration increased, probably due to the presence of deionized water inside the capsule, since no metal adsorption by cellulose membrane occurred (data in S1 Table). Unlike the free biosorbent, which has a limited number of binding sites, deionized water inside the capsules can also remove copper ions from the external solution because copper ions permeate the membrane by passive diffusion until reaching equilibrium. Then, the total biosorption capacity of the capsules can be considered as the sum of the copper ions that are adsorbed by fungal biomass and the ions that are removed by the liquid phase inside the capsule. As shown in the desorption experiments, these ions in this liquid phase can be removed and recovered along with the ions adsorbed by the biosorbent.

In order to demonstrate the water contribution inside the capsule in removing copper ions in the total biosorption capacity, the Eq 1 was reformulated to calculate the adsorption of copper ions only by the biosorbent inside the capsule, as shown below (Eq 9):

$$q\;(mg.g^{-1})=\frac{\left(C_{0}.V_{0})-(C_{f}.V_{f}\right)}{m}$$ (9)

where m (g) is the mass of the biosorbent, V_0_ (L) is the volume of the external solution to the capsule, C_0_ (mg L^-1^) is the initial solution copper concentration, V_f_ (L) is the sum of the volume of the external and internal solution of the capsule and C_f_ (mg L^-1^) is the solution copper concentration at the time of sampling. The value of q (mg g^-1^) is expressed in milligrams of metal adsorbed per gram of biosorbent.

In this equation, the amount of ions adsorbed on the biomass inside the capsule was obtained by the difference between of the initial amount of metal ions (*C*_0_. *V*_0_) and the amount of ions remaining in solution (inside and outside the capsule), which not adsorbed on the biomass (*C_f_*. *V_f_*). With this equation was possible to calculate the adsorption capacity of the biomass enclosed within the capsule, disregarding the removal of copper ions by aqueous phase in the encapsulated biosorbents. Thus, EB10x and EB30x were used to designate copper adsorption only by the biosobent inside the capsule.

**Fig 4:**
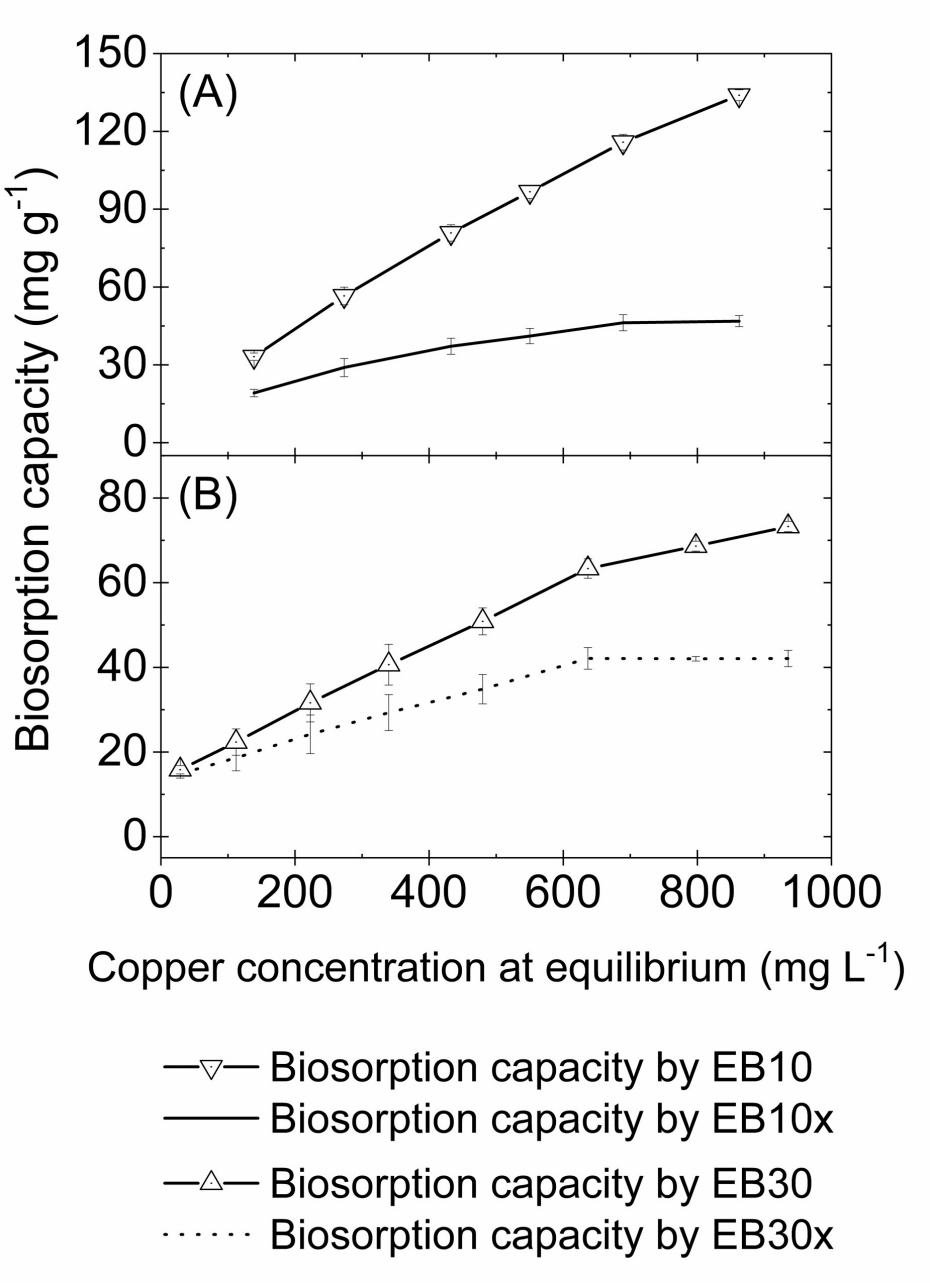
Biosorption capacity of copper by EB10 and EB10x (A) and EB30 and EB30x (B). Conditions: Initial copper concentrations ranging from about 50 to 1050 mg L^-1^; contact time = 240 min; pH = 5.0; agitation speed = 150 rpm; temperature = 25 ± 2°C; biosorbent dosage for EB10 = 0.333 g L^-1^ and for EB30 = 1.0 g L^-1^.

The biosorption isotherms by EB10, EB30, EB10x and EB30x are shown in Fig 4:

As Fig 4 shows, when copper removal by the aqueous phase in the capsule is disregarded (Eq 9), it can be observed that the biosorption attained the equilibrium plateau, indicating that all available sites of the encapsulated biosorbents have been saturated. In this plateau, the maximum experimental adsorption capacity of the EB30x and EB10x can be obtained, which are 42.1 ± 2.5 mg g^-1^ and 46.2 ± 3.1 mg g^-1^, respectively. These values are lower than the biosorption capacities of the EB10 and EB30 that consider the removal of ions by the entire capsule (ions removed by the aqueous phase and biomass inside the capsule), which are 63.3 ± 2.3 mg g^-1^ for EB30 and 115.9 ± 3.0 mg g^-1^ for EB10, thus at this point the contribution of the aqueous phase inside the capsules in the biosorptive capacity corresponds to 21.3 mg g^-1^ (33.6%) and 69.6 mg g^-1^ (60.1%), respectively. In addition, even after all of the biomass active sites are saturated, as indicated by the plateau obtained for EB10x and EB30x (Fig 4), the aqueous phase inside the capsule can continue removing ions from the external environment. These results suggest an important synergy between the liquid and solid phases inside the capsule resulting in increased metal removal. This synergy can be explained not only by the difference in metal concentration ions between the two aqueous phases separated by the semipermeable membrane, but also due to the excess of negative charges of the organic biomass functions, which cannot cross the membrane. The accumulation of negative charges inside the capsule, which cannot penetrate all the phases of the system (biomass, internal liquid and external liquid phases), leads to a difference in electrical potential between the internal and external phases of the capsule [68]. This difference in electrical potential can attract and concentrate metal ions, which are positively charged, inside the capsule so that the electrochemical system can achieve electrochemical equilibrium.

In this study, Langmuir and Freundlich models were applied to the experimental biosorption data (data in S2 Table). These isotherms are important to describe the relationship between the amount of ions removed per gram of biosorbent (q_eq_), its equilibrium concentration (C_eq_), as well as to determine essential parameters for practical adsorption operation, which are summarized in Table 2.

**Table 2:** Parameters of Langmuir and Freundlich isotherms for copper biosorption by EB30x, EB10x, EB30 and EB10. Conditions: Initial copper concentrations ranging from about 50 to 1050 mg L^-1^; contact time = 240 min; pH = 5.0 ± 0.1; agitation speed = 150 rpm; temperature = 25 ± 2°C; biosorbent dosage for EB10 = 0.333 g L^-1^ and for EB30 = 1.0 g L^-1^.

Isothermal Models	Parameters	EB30x	EB10x	EB30	EB10
**Freundlich**	k_F_ (mg^1-1/n^ L^1/n^ g^-1^)	2.60	1.63	1.41	0.751
	1/n (L g^-1^)	0.417	0.508	0.581	0.770
	r^2^	0.977	0.981	0.995	1.00
	RMSD	1.84	1.81	1.53	1.12
**Langmuir**	k_L_ (L mg^-1^)	0.0039	0.0029	0.0018	0.0008
	q_max_ (mg g^-1^)	54.8	67.2	116.1	333.5
	r^2^	0.984	0.994	0.968	0.980
	RMSD	1.70	0.804	2.11	1.47

As can be seen in Table 2, the EB30x and EB10x biosorption was better adjusted by the Langmuir isotherm based on the values of r^2^ and RMSD. According to the Langmuir adsorption model, we can assume that the copper ion adsorption by biomass inside the capsules predominantly occurs in monolayers with homogeneous binding sites and constant adsorption energy [53, 69]. In contrast, copper adsorption data by EB10 and EB30 fitted well to the Freundlich isotherm, as demonstrated by the r^2^ values (0.99) close to 1.0 and lower RMSD (1.12 and 1.53 to EB10 and EB30, respectively) compared to those of Langmuir (Table 2). Here, the copper removal by encapsulated biosorbents considered both ions adsorbed by the biomass and those that diffused through the membrane and remained in the aqueous phase inside the capsules. The Freundlich model is widely used to describe the adsorption onto heterogeneous sorption sites with non-uniform distribution of the heat of sorption and affinities [70]. In addition, this model describes the distribution of metal ions between solid and aqueous phases at the point of saturation [53].

Despite the limitation of applying the Langmuir model for copper removal by EB10 and EB30 capsules due to the contribution of the aqueous phase in its biosorption capacity, the experimental equilibrium biosorption data also showed a good fit to the Langmuir isotherms (r^2^ > 0.96). Thus, the maximum capacity for EB10 and EB30 could be estimated, whose values were 333.5 and 116.1 mg g^-1^, respectively (Table 2).

According to the biosorption capacity values (Table 2), the EB10 and EB10x capsules presented a higher biosorption capacity than the EB30 and EB30x capsules. Based on these results, it can be suggested that the higher the biosorbent concentrations, which were 30 g L^-1^ for EB30 and EB30x and 10 g L^-1^ for EB10 and EB10x, the lower the biosorption capacity. This may explain the fact that free biomass, with 1g L^-1^ of biosorbent concentration, has shown greater biosorption capacity than EB30x and EB10x. Indeed, it has been reported in other studies that in higher biosorbent concentrations, there is a decrease in the surface area available for adsorption due to electrostatic interactions between the functional groups on the biosorbent surfaces, which decrease the biosorption capacity [45, 69, 71].

Comparing the q_max_ data to other immobilized biosorbents described in the literature (Table 3), the EB10 capsules displayed the highest maximum copper biosorption capacity, indicating its potential for copper removal from the aqueous solution as industrial wastewater.

**Table 3:** Comparison between the maximum biosorption capacities (q_max_) predicted by the Langmuir model for copper ions by encapsulated biosorbent (EB10 and EB30) in relation to other immobilized fungal biosorbents.

Biosorbent	Methods of immobilization	q_max_ (mg g^-1^)	Equilibrium time (min.)	pH	T(°C)	Ref.
*Trametes versicolor*	Carboxymethyl cellulose	124.6	60	6	25	[72]
*Phanerochaete chrysosporium*	Loofah Sponge	98.9	60	6	20	[73]
*Penicillium simplicissimum*	Loofah Sponge	106.4	60	5	30	[74]
*Pycnoporus sanguineus*	Sodium alginate	2.77	180	5	30	[71]
*Aspergillus niger*	Polyvinyl alcohol	34.1	25	5.5	30	[46]
*Penicillium simplicissimum*	Zeolite	30.7	180	3	-	[57]
*Trichoderma asperellum*	Sodium alginate	140.9	240	5	30	[75]
*Penicillium janthinillum*	PVA with sodium alginate	13.6	100	-	32	[35]
*Candida krusei*	Calcium alginate	153.7	60	5.2	30	[43]
*Aspergillus nidulans*	Cellulose membrane capsule (EB30)	116.1	240	5	25	Present work
*Aspergillus nidulans*	Cellulose membrane capsule (EB10)	333.5	240	5	25	Present work

### Biosorption mechanism

Since biosorption takes place essentially in the cell wall, the mechanism by which fungi adsorb metal ions from aqueous solution depends on the surface properties of the biomass [14]. Different mechanisms, including electrostatic interactions, complexation/coordination, precipitation and ion exchange, are involved in biosorption phenomenon [3, 8]. Therefore, the knowledge of the cell wall composition of the fungal biomass is of great importance for the efficiency of biosorption process [76].

Fungal cell walls are composed mostly of polysaccharides (glucans, chitin and chitosan, mannans and/or galactomannans) and glycoproteins, lipids, pigments are in lower proportions. Various functional groups, including amine, carboxyl, phosphate, sulfate, sulfhydryl and hydroxyl, present in these polymers offer a large number of metal ion binding sites [54]. In order to identify the functional groups present in the *A*. *nidulans* biomass surface and to elucidate the mechanism involved in copper binding, we performed the FT-IR analysis of metal-free biomass (before biosorption) and those loaded with copper (after biosorption).

**Fig 5:**
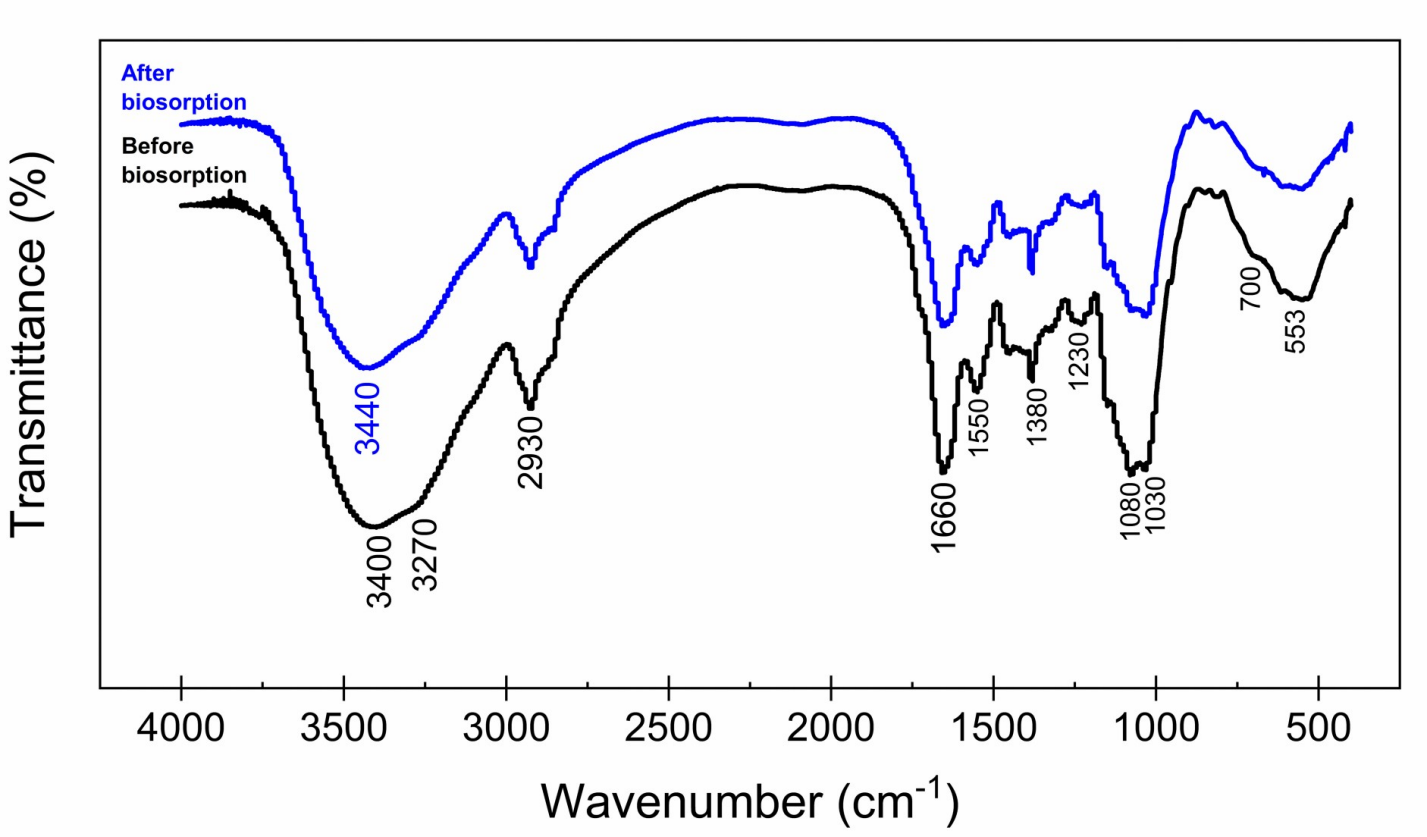
FT-IR spectra of fungal biomass before and after copper biosorption.

As shown in Fig 5, the FT-IR spectra before and after biosorption were more similar, but some changes in the profile were observed due to the copper adsorption. Both spectra have a strong and broad band in the region between 3650–3140 cm^-1^, which can be attributed to the axial stretching of the hydroxyl (–OH) or amine (–NH_2_ and/or R_2_NH) groups. However, after copper biosorption, there was a decrease in the intensity of these bands and a peak shift from 3400 to 3440 cm^-1^, indicating possible chemical interactions between copper ions and the hydroxyl or amine groups present on the biomass. For the peak at 2930 cm^-1^, attributed to the axial stretch of the C–H bond, as well as that at 1380 cm^-1^, due to angular deformation of the O–H bond, no change was observed after copper biosorption.

It is also possible to observe in Fig 5, the peaks at 1660 cm^-1^, assigned to C = O stretching vibration in carboxyl groups and at 1550 cm^-1^, attributed to the–CN stretching vibration and/or–NH bending vibration, were slightly reduced after biosorption, which may be indicative that copper ions interact with these groups in the biomass by electrostatic attraction. Wan et al. [77] also reported that the −CN stretching and C = O stretching groups were primary sorption sites for copper binding.

For peaks in the 1200–1000 cm^-1^ region, attributed to the C–O stretching, there was a marked reduction in the intensity of the band after biosorption, indicating that alcohols, and carboxylic acids from the biomass participate in the adsorption of copper ions. In addition, the peaks observed at 700 and 533 cm^-1^ was also reduced, which can be attributed to the stretching vibration of the Cu–O bond in biomass (Fig 5). Based on these results, we can suggest that the–NH,–CH,–OH, C = O and C–O groups on the biomass surface participate in the copper biosorption process. The presence of these groups is due to the *A*. *nidulans* biomass used in this study having the DOPA-melanin pigment containing several functional groups [29], which may act as potential metal binding sites. These results are in agreement with our previous studies, which demonstrated that the pigmented biomass produced by the MEL1 mutant from *Aspergillus nidulans* possesses the DOPA-melanin containing several functional groups, e.g., carboxyl, phenolic and alcoholic hydroxyl [29], which may act as the main metal binding sites, increasing the biomass adsorption capacity. The presence of these negatively charged functional groups on the cell wall of the fungal biomass facilities the interaction with the positive charges of copper ions via electrostatic attraction. This interaction of metal with the surface ligands of biomass provides the adsorption process, holding copper ions inside the pores of the biomass [2, 18]. Similar results are described in others studies reported in the literature [76–80].

Moreover, biosorption may also involve the formation of copper complexes on the biomass surface, which increase the biosorption capacity [81]. The Eq 10 describes the possible coordination mechanism of copper with the oxygen present in the functional groups of *A*. *nidulans* biomass:

$$SBS-OH+Cu^{2+}⇌SBS-OCu^{+}+H^{+}$$ (10)

where SBS represents the surface binding site.

As can be seen from Eq 10, the ion exchange between H^+^ and Cu^2+^ leads to proton release, which explains the decrease in pH observed during biosorption kinetics (when there was no pH adjustment during the process), indicating that the ion exchange mechanism is also involved in copper adsorption by fungal biomass.

**Fig 6:**
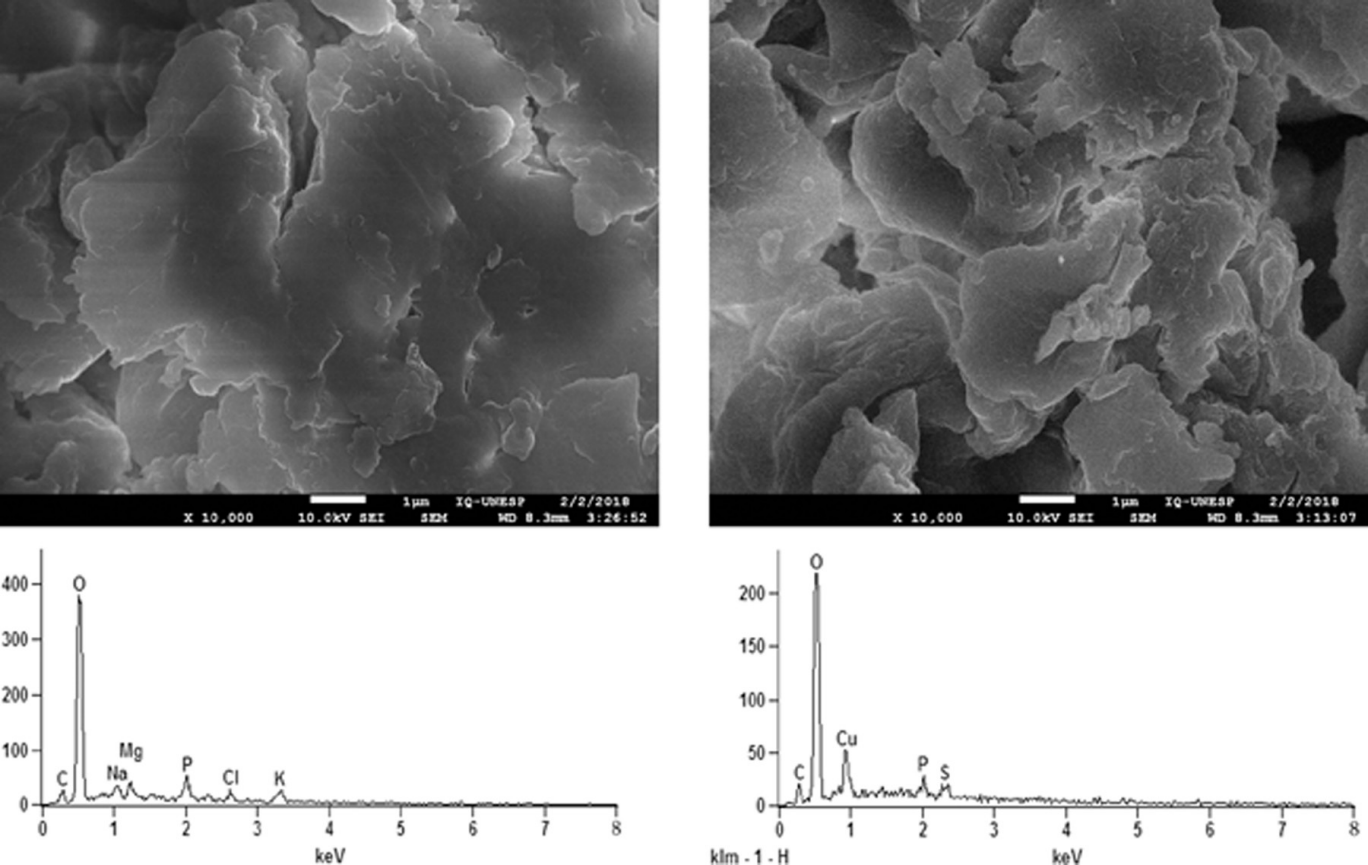
SEM images and EDXS spectra of fungal biomass. Photomicrograph of the surface of biomass before (A) and after (C) copper biosorption. Elementary analysis by EDXS of biomass before (B) and after (D) exposure to the metal.

In this study, the analysis of the changes on the surface fungal biomass, as well as its elemental composition, before and after the biosorption process, using Scanning Electron Microscopy (SEM) equipped with Energy Dispersive X-ray Spectroscopy (EDXS) are shown in Fig 6.

No difference was observed in the morphology of the biomass surfaces before and after the biosorption process (Fig 6A and 6C). However, the qualitative elementary analyses by EDXS demonstrate the appearance of a strong copper signal in the biosorbent after biosorption (Fig 6D), confirming the binding copper in the fungal biomass. In addition, the Na, K and Mg signals disappeared after biosorption (Fig 6D), indicating possible binding sites for copper ions. This result confirmed that the binding of copper to the biosorbent may be explained by the ion-exchange mechanism between species previously bound in functional groups of the fungal cell wall and the copper ions present in the solution. Yuncu, Sanin and Yetis [82] also observed a concomitant Ca^2+^ and Mg^2+^ release with sorption and suggested that ion exchange plays an important role in heavy metal biosorption but it was not the only mechanism involved during the sorption process.

Thereby, based on FT-IR and SEM-EDXS analysis, it is possible to suggest that ion-exchange, coordination and electrostatic attraction mechanisms seem to be involved in copper adsorption by *A*. *nidulans* biomass, and these mechanisms were largely reversible, as shown by desorption studies.

### Desorption studies

The industrial biosorption application for heavy metal removal from aqueous solution depends on the metal recovery efficiency [5]. The choice of the agent and concentration used for metal desorption must consider minimal damage caused to the biosorbent physical properties, so that its metal bonding efficiency remains in its original state to ensure its maximum efficiency for metal binding [40]. In this study, the desorption of copper of the EB30 capsule performed using different desorbent concentrations showed that the copper recovery efficiencies were 80.4%, 77.1% and 76.4% for HCl concentrations of 0.05, 0.1 and 0.2 mol L^-1^, respectively. Other studies also showed that the amount of copper ions desorbed gradually decreased as the HCl concentration increased and metal recoveries may be higher at lower concentrations of the desorbent agent [9, 83].

**Fig 7:**
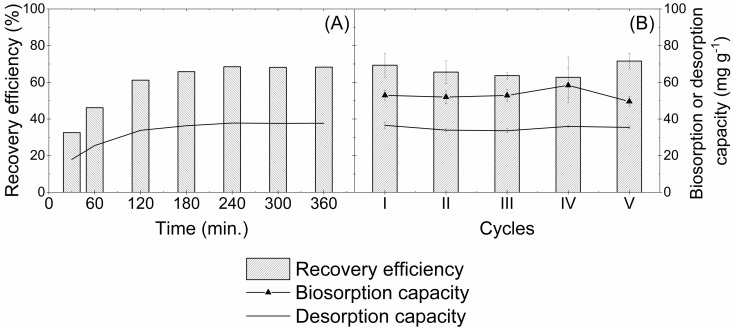
Desorption kinetics (A), biosorption/desorption capacity and copper recovery efficiency in successive cycles (B) by EB30. Conditions: Initial copper concentration = 750 mg L^-1^; pH = 5.0; biosorbent dosage for EB30 = 1.0 g L^-1^, desorbing agent = HCl (0.05 mol L^-1^); contact time of 240 min; agitation speed = 150 rpm; temperature = 25 ± 2°C.

The copper desorption kinetics from EB30 using 0.05 mol L^-1^ of HCl showed that copper recovery increased more rapidly in the initial stages until reaching equilibrium in approximately 240 min with a recovery efficiency of approximately 70% (Fig 7A). This equilibrium time is the same for the biosorption equilibrium (Fig 2), suggesting that the diffusion of ions through the membrane is the rate-limiting step of the process.

In order to evaluate the biosorbent reusability for copper ion removal, five successive biosorption-desorption cycles were performed using the same batch of encapsulated biosorbent (EB30 capsule). As shown in Fig 7, there was no decrease in biosorption or desorption capacities during the cycles. The recovery efficiency after the five consecutive biosorption/desorption cycles reached 71.6% (Fig 7). Compared to the literature data [72, 74, 75], the high recovery efficiency displayed by the encapsulated biosorbent indicates its potential to remove and recover copper ions in consecutive biosorption/desorption cycles from aqueous solution.

## Conclusions

According to the results obtained, the maximum biosorption capacities for the encapsulated biosorbent were higher (333.5 and 116.1 mg g^-1^ for capsules containing 10 and 30 mg of biomass mL^-1^ deionized water, respectively) compared to free biomass (92.0 mg g^-1^) and its metal recovery efficiency remained constant at approximately 70% during five biosorption/desorption cycles. Therefore, this study demonstrated that the new encapsulation method of the highly pigmented biomass from *Aspergillus nidulans* mutant using a semipermeable cellulose membrane is efficient for metal removal and recovery in multiple adsorption-desorption cycles. In addition, this reversible encapsulation method can be considered an attractive strategy for large-scale metal removal and recovery from industrial wastewaters mainly due to its low cost and the possibility of recovering adsorbed ions and the reuse of biosorbent in consecutive biosorption/desorption cycles with high efficiency of metal recovery.

## Supporting information

S1 Appendix The estimated regression coefficients, standard error, p-values, confidence intervals and measures of goodness of fit of the kinetic and adsorption models applied. (XLSX)

S1 Table The biosorption capacity of copper (q) from the pseudo-first and pseudo-second order models for encapsulated biosorbents (EB10 and EB30) and control as a function of the biosorption time. (DOCX)

S2 Table The biosorption capacity of copper (q) from Langmuir and Freundlich models for encapsulated biosorbents (EB10, EB10x, EB30 and EB30x) and free biomass at different copper concentrations at equilibrium. (DOCX)

S3 Table Desorption capacity (q_des_) and recovery efficiency of copper by the encapsulated biosorbent (EB30) as a function of desorption time. (DOCX)

S4 Table Biosorption (q) and desorption (q_des_) capacity and recovery efficiency of copper by the encapsulated biosorbent (EB30) as a function of successive cycles. (DOCX)

## References

[1] Sethurajan M, Lens PNL, Horn HA, Figueiredo LHA, Hullebusch ED van. Leaching and Recovery of Metals. Rene ER, Sahinkaya E, Lewis A, Lens PNL, editors. Sustainable Heavy Metal Remediation. Cham: Springer International Publishing; 2017. p. 161–206. doi: 10.1016/j.jhazmat.2016.01.028

[2] Singh S, Kumar V, Datta S, Dhanjal DS, Sharma K, Samuel J, et al. Current advancement and future prospect of biosorbents for bioremediation. Sci Total Environ. 2020;709. pii: S0048969719358905. doi: 10.1016/j.scitotenv.2019.135895 31884296

[3] Bashir A, Malik LA, Ahad S, Manzoor T, Bhat MA, Dar GN, et al. Removal of heavy metal ions from aqueous system by ion-exchange and biosorption methods. Environ Chem Lett. 2019;17(2):729–54.

[4] Stern BR, Solioz M, Krewski D, Aggett P, Aw T-C, Baker S, et al. Copper and Human Health: Biochemistry, Genetics, and Strategies for Modeling Dose-response Relationships. J Toxicol Environ Heal Part B. 2007 Apr 3;10(3):157–222. doi: 10.1080/10937400600755911 17454552

[5] Qin H, Hu T, Zhai Y, Lu N, Aliyeva J. The improved methods of heavy metals removal by biosorbents: A review. Environ Pollut. 2020 Mar;258. pii: S0269749119351218?via%3Dihub. doi: 10.1016/j.envpol.2019.113777 31864928

[6] Rahman Z, Singh VP. Bioremediation of toxic heavy metals (THMs) contaminated sites: concepts, applications and challenges. Environ Sci Pollut Res. 2020 Aug 16;27(22):27563–81. doi: 10.1007/s11356-020-08903-0 32418096

[7] Coelho LMM, Rezende HC, Coelho LMM, de Sousa PAR, Melo DFO, Coelho NMM. Bioremediation of Polluted Waters Using Microorganisms. In: Shiomi N, editor. Advances in Bioremediation of Wastewater and Polluted Soil [Internet]. InTech; 2015. p. 1–22. Available from: http://www.intechopen.com/books/advances-in-bioremediation-of-wastewater-and-polluted-soil/bioremediation-of-polluted-waters-using-microorganisms

[8] Fomina M, Gadd GM. Biosorption: current perspectives on concept, definition and application. Bioresour Technol. 2014 May;160:3–14. doi: 10.1016/j.biortech.2013.12.102 24468322

[9] Ojima Y, Kosako S, Kihara M, Miyoshi N, Igarashi K, Azuma M. Recovering metals from aqueous solutions by biosorption onto phosphorylated dry baker’s yeast. Sci Rep. 2019 Dec 18;9(1):225. pii: s41598-018-36306-2. doi: 10.1038/s41598-018-36306-2 30659210 PMC6338781

[10] Shamim S. Biosorption of Heavy Metals. In: Derco J, Vrana B, editors. Biosorption. InTech; 2018. p. 21–49.

[11] Vijayaraghavan K, Balasubramanian R. Is biosorption suitable for decontamination of metal-bearing wastewaters? A critical review on the state-of-the-art of biosorption processes and future directions. J Environ Manage. 2015 Sep;160:283–96. doi: 10.1016/j.jenvman.2015.06.030 26143501

[12] Wu M, Liang J, Tang J, Li G, Shan S, Guo Z, et al. Decontamination of multiple heavy metals-containing effluents through microbial biotechnology. J Hazard Mater. 2017 Sep;337:189–97. doi: 10.1016/j.jhazmat.2017.05.006 28521206

[13] Alothman ZA, Bahkali AH, Khiyami MA, Alfadul SM, Wabaidur SM, Alam M, et al. Low cost biosorbents from fungi for heavy metals removal from wastewater. Sep Sci Technol. 2020 Jul 2;55(10):1766–75.

[14] Dusengemungu L, Kasali G, Gwanama C, Ouma KO. Recent Advances in Biosorption of Copper and Cobalt by Filamentous Fungi. Front Microbiol. 2020 Dec 21;11(December):0–16. 10.3389/fmicb.2020.582016 PMC7779407 33408701

[15] Lo YC, Cheng CL, Han YL, Chen BY, Chang JS. Recovery of high-value metals from geothermal sites by biosorption and bioaccumulation. Bioresour Technol. 2014;160:182–90. doi: 10.1016/j.biortech.2014.02.008 24581863

[16] Vieira RHSF, Volesky B. Biosorption: a solution to pollution? Int Microbiol. 2000 Mar;3(1):17–24. 10963329

[17] Gupta VK, Nayak A, Agarwal S. Bioadsorbents for remediation of heavy metals: Current status and their future prospects. Environ Eng Res. 2015 Mar 31;20(1):1–18.

[18] Kumar V, Dwivedi SK. Mycoremediation of heavy metals: processes, mechanisms, and affecting factors. Environ Sci Pollut Res. 2021 Mar 6;28(9):10375–412. doi: 10.1007/s11356-020-11491-8 33410020

[19] Li Y, Zou G, Yang S, Wang Z, Chen T, Yu X, et al. Integration of bio-inspired adsorption and photodegradation for the treatment of organics-containing radioactive wastewater. Chem Eng J. 2019 May;364(January):139–45.

[20] Lu N, Hu T, Zhai Y, Qin H, Aliyeva J, Zhang H. Fungal cell with artificial metal container for heavy metals biosorption: Equilibrium, kinetics study and mechanisms analysis. Environ Res. 2020 Mar;182(September 2019). pii: S0013935119308588?via%3Dihub. doi: 10.1016/j.envres.2019.109061 31901626

[21] Mosa KA, Saadoun I, Kumar K, Helmy M, Dhankher OP. Potential Biotechnological Strategies for the Cleanup of Heavy Metals and Metalloids. Front Plant Sci. 2016 Mar 15;7(MAR2016):1–14. doi: 10.3389/fpls.2016.00001 27014323 PMC4791364

[22] Kapoor A, Viraraghavan T, Cullimore DR. Removal of heavy metals using the fungus *Aspergillus niger*. Bioresour Technol. 1999 Oct;70(1):95–104.

[23] Volesky B. Biosorption and me. Water Res. 2007 Oct;41(18):4017–29. doi: 10.1016/j.watres.2007.05.062 17632204

[24] Tatiana A Belozerskaya, Natalya N. Gessler A, Aver. Melanin Pigments of Fungi. Mérillon J-M, Ramawat KG, editors. Fungal Metabolites. Cham: Springer International Publishing; 2017. p. 263–292.

[25] Gow NAR, Latge J-P, Munro CA. The Fungal Cell Wall: Structure, Biosynthesis, and Function. In: Heitman J, Howlett BJ, Crous PW, Stukenbrock EH, James TY, Gow NAR, editors.The Fungal Kingdom. Washington, DC, USA: ASM Press; 2017. p. 267–92.

[26] Nosanchuk JD, Stark RE, Casadevall A. Fungal Melanin: What do We Know About Structure? Front Microbiol. 2015 Dec 22;6(DEC):1–7. doi: 10.3389/fmicb.2015.01463 26733993 PMC4687393

[27] Pombeiro-Sponchiado SR, Sousa GS, Andrade JCR, Lisboa HF, Gonçalves RCR. Production of Melanin Pigment by Fungi and Its Biotechnological Applications. In: Blumenberg M, editor. Melanin. InTech; 2017. p. 47–75. Available from: https://www.intechopen.com/books/melanin/production-of-melanin-pigment-by-fungi-and-its-biotechnological-applications

[28] Caporalin CB. Comparação da biossorção de metais terras-raras pela biomassa melanizada do fungo *Aspergillus nidulans* nas formas livre e imobilizada [dissertation]. Universidade Estadual Paulista; 2011. Available from: https://repositorio.unesp.br/handle/11449/88024

[29] Gonçalves RCR, Lisboa HCF, Pombeiro-Sponchiado SR. Characterization of melanin pigment produced by *Aspergillus nidulans*. World J Microbiol Biotechnol. 2012 Apr 16;28(4):1467–74. doi: 10.1007/s11274-011-0948-3 22805928

[30] Cordero RJB, Casadevall A. Functions of fungal melanin beyond virulence. Fungal Biol Rev. 2017 Mar;31(2):99–112. doi: 10.1016/j.fbr.2016.12.003 31649746 PMC6812541

[31] Manirethan V, Balakrishnan RM. Batch and continuous studies on the removal of heavy metals using biosynthesised melanin impregnated activated carbon. Environ Technol Innov. 2020 Nov;20. pii: S2352186420313857. doi: 10.1016/j.eti.2020.101085 31602598

[32] Nakkeeran E, Rathna R, Viveka R. Mechanism and Action of *Aureobasidium pullulans* on Biosorption of Metals. In: Varjani S., Gnansounou E., Gurunathan B., Pant D. ZZ, editor. Waste Bioremediation Energy, Environment, and Sustainability. Singapore: Springer; 2018. p. 215–31.

[33] Thaira H, Raval K, Manirethan V, Balakrishnan RM. Melanin nano-pigments for heavy metal remediation from water. Sep Sci Technol. 2019 Jan 22;54(2):265–74.

[34] Tran-Ly AN, Ribera J, Schwarze FWMR, Brunelli M, Fortunato G. Fungal melanin-based electrospun membranes for heavy metal detoxification of water. Sustain Mater Technol. 2020 Apr;23. pii: S2214993719301228. doi: 10.1016/j.susmat.2019.e00146

[35] Cai C-X, Xu J, Deng N-F, Dong X-W, Tang H, Liang Y, et al. A novel approach of utilization of the fungal conidia biomass to remove heavy metals from the aqueous solution through immobilization. Sci Rep. 2016 Dec 16;6(1): 36546. Available from: 10.1038/srep36546. PMC5111076 27848987

[36] Cheng Y, Zhou F, Li S, Chen Z. Removal of mixed contaminants, crystal violet, and heavy metal ions by using immobilized stains as the functional biomaterial. RSC Adv. 2016;6(72):67858–65.

[37] Chen B-Y, Chen C-Y, Guo W-Q, Chang H-W, Chen W-M, Lee D-J, et al. Fixed-bed biosorption of cadmium using immobilized *Scenedesmus obliquus* CNW-N cells on loofa (*Luffa cylindrica*) sponge. Bioresour Technol. 2014 May;160:175–81. doi: 10.1016/j.biortech.2014.02.006 24581862

[38] Zhang T, Wang Y, Kuang Y, Yang R, Ma J, Zhao S, et al. Adsorptive removal of Cr^3+^ from aqueous solutions using chitosan microfibers immobilized with plant polyphenols as biosorbents with high capacity and selectivity. Appl Surf Sci. 2017 May;404:418–25.

[39] Ghazvini PTM, Mashkani SG. Effect of salinity on vanadate biosorption by *Halomonas sp*. GT-83: Preliminary investigation on biosorption by micro-PIXE technique. Bioresour Technol. 2009 Apr;100(8):2361–8. doi: 10.1016/j.biortech.2008.11.025 19117752

[40] Giese EC, Silva DD V., Costa AFM, Almeida SGC, Dussán KJ. Immobilized microbial nanoparticles for biosorption. Crit Rev Biotechnol. 2020 Jul 3;40(5):653–66. doi: 10.1080/07388551.2020.1751583 32299253

[41] He S, Ruan B, Zheng Y, Zhou X, Xu X. Immobilization of chlorine dioxide modified cells for uranium absorption. J Environ Radioact. 2014 Nov;137:46–51. doi: 10.1016/j.jenvrad.2014.06.016 24998748

[42] Kumari S, Mahapatra S, Das S. Ca-alginate as a support matrix for Pb(II) biosorption with immobilized biofilm associated extracellular polymeric substances of *Pseudomonas aeruginosa* N6P6. Chem Eng J. 2017 Nov;328:556–66.

[43] Luk CHJ, Yip J, Yuen CWM, Pang SK, Lam KH, Kan CW. Biosorption Performance of Encapsulated *Candida krusei* for the removal of Copper(II). Sci Rep. 2017 Dec 19;7(1):2159. Available from: doi: 10.1038/s41598-017-02350-7 28526881 PMC5438343

[44] Migahed F, Abdelrazak A, Fawzy G. Batch and continuous removal of heavy metals from industrial effluents using microbial consortia. Int J Environ Sci Technol. 2017 Jun;14(6):1169–80.

[45] Ramteke LP, Gogate PR. Treatment of water containing heavy metals using a novel approach of immobilized modified sludge biomass based adsorbents. Sep Purif Technol. 2016 May;163:215–27.

[46] Tsekova K, Todorova D, Dencheva V, Ganeva S. Biosorption of copper(II) and cadmium(II) from aqueous solutions by free and immobilized biomass of *Aspergillus niger*. Bioresour Technol. 2010 Mar;101(6):1727–31. doi: 10.1016/j.biortech.2009.10.012 19906526

[47] Vijayaraghavan K, Yun Y-S. Bacterial biosorbents and biosorption. Biotechnol Adv. 2008 May;26(3):266–91. doi: 10.1016/j.biotechadv.2008.02.002 18353595

[48] Sponchiado SRP, Sousa GS, Lisboa HCF, Golçalves RCR, inventors; Universidade Estadual Paulista Júlio de Mesquita Filho, assignee. Processo de produção do pigmento melanina pelo fungo *Aspergillus nidulans*. Brazil patent BR1020180079522A2. 2018 Apr 19.

[49] Lagergren S. Zur theorie der sogenannten adsorption gelöster stoffe, Kungliga Svenska Vetenskapsakademiens. Handlingar. 1898;24(4):1–39.

[50] Febrianto J, Kosasih AN, Sunarso J, Ju Y-H, Indraswati N, Ismadji S. Equilibrium and kinetic studies in adsorption of heavy metals using biosorbent: A summary of recent studies. J Hazard Mater. 2009 Mar;162(2–3):616–45. doi: 10.1016/j.jhazmat.2008.06.042 18656309

[51] Langmuir I. The adsorption of gases on plane surfaces of glass, mica and platinum. J Am Chem Soc. 1918 Sep 1;40(9):1361–403.

[52] Freundlich HMF. Over the Adsorption in Solution. J Phys Chem. 1906;57:385–471.

[53] Beni AA, Esmaeili A. Biosorption, an efficient method for removing heavy metals from industrial effluents: A Review. Environ Technol Innov. 2020 Feb;17. pii: S2352186418304346. doi: 10.1016/j.eti.2019.100503

[54] Dhankhar R, Hooda A. Fungal biosorption–an alternative to meet the challenges of heavy metal pollution in aqueous solutions. Environ Technol. 2011 Apr;32(5):467–91. doi: 10.1080/09593330.2011.572922 21877528

[55] Ho YS, McKay G. A Comparison of Chemisorption Kinetic Models Applied to Pollutant Removal on Various Sorbents. Process Saf Environ Prot. 1998 Nov;76(4):332–40.

[56] Ho YS, Ng JCY, McKay G. Kinetics of pollutant sorption by biosorbents: review. Sep Purif Methods. 2000 Oct 7;29(2):189–232.

[57] Bakatula EN, Cukrowska EM, Straker CJ, Weiersbye IM, Tutu H. Biosorption of metals from gold mine wastewaters by *Penicillium simplicissimum* immobilized on zeolite: Kinetic, equilibrium and thermodynamic studies. In: Rüde TR, Freund A, Wolkersdorfer C, editors. 11th International Mine Water Association Congress: Mine Water–Managing the Challenges. Aachen; 2011. p. 271–5.

[58] Hasan SH, Srivastava P. Batch and continuous biosorption of Cu^2+^ by immobilized biomass of *Arthrobacter sp*. J Environ Manage. 2009 Aug;90(11):3313–21. doi: 10.1016/j.jenvman.2009.05.005 19487070

[59] Lee H, Shim E, Yun H-S, Park Y-T, Kim D, Ji M-K, et al. Biosorption of Cu(II) by immobilized microalgae using silica: kinetic, equilibrium, and thermodynamic study. Environ Sci Pollut Res. 2016 Jan 9;23(2):1025–34. 10.1007/s11356-015-4609-1 25953610

[60] Xie H, Zhao Q, Zhou Z, Wu Y, Wang H, Xu H. Efficient removal of Cd(II) and Cu(II) from aqueous solution by magnesium chloride-modified *Lentinula edodes*. RSC Adv. 2015;5(42):33478–88.

[61] Netzahuatl-Muñoz AR, Cristiani-Urbina M del C, Cristiani-Urbina E. Chromium Biosorption from Cr(VI) Aqueous Solutions by *Cupressus lusitanica* Bark: Kinetics, Equilibrium and Thermodynamic Studies. Tajmir-Riahi H-A, editor. PLoS One. 2015 Sep 9;10(9): e0137086. Available from: doi: 10.1371/journal.pone.0137086 PMC4564179 26352933

[62] Alp Arıcı T, Özcan AS, Özcan A. Biosorption Characteristics of Cu(II) and Cd(II) Ions by Modified Alginate. J Polym Environ. 2020 Dec 6;28(12):3221–34.

[63] Barquilha CER, Cossich ES, Tavares CRG, Silva EA. Biosorption of nickel(II) and copper(II) ions in batch and fixed-bed columns by free and immobilized marine algae *Sargassum sp*. J Clean Prod. 2017 May;150:58–64.

[64] Dursun AY. A comparative study on determination of the equilibrium, kinetic and thermodynamic parameters of biosorption of copper(II) and lead(II) ions onto pretreated *Aspergillus niger*. Biochem Eng J. 2006 Feb;28(2):187–95.

[65] El-Morsy E-SM. *Cunninghamella echinulata* a new biosorbent of metal ions from polluted water in Egypt. Mycologia. 2004 Nov 30;96(6):1183–9. 21148940

[66] Fawzy MA. Biosorption of copper ions from aqueous solution by *Codium vermilara*: Optimization, kinetic, isotherm and thermodynamic studies. Adv Powder Technol. 2020 Sep;31(9):3724–35.

[67] Sheng PX, Ting Y-P, Chen JP. Biosorption of Heavy Metal Ions (Pb, Cu, and Cd) from Aqueous Solutions by the Marine Alga *Sargassum sp*. in Single- and Multiple-Metal Systems. Ind Eng Chem Res. 2007 Apr;46(8):2438–44.

[68] Levine IN. Electrochemical Systems. In: Physical Chemistry. 6th ed. New York: McGraw-Hill; 2009.

[69] Kapoor A. Fungal biosorption—an alternative treatment option for heavy metal bearing wastewaters: a review. Bioresour Technol. 1995;53(3):195–206.

[70] Freundlich H, Heller W. The Adsorption of cis—and trans -Azobenzene. J Am Chem Soc. 1939 Aug;61(8):2228–30.

[71] Yahaya YA, Mat Don M, Bhatia S. Biosorption of copper (II) onto immobilized cells of *Pycnoporus sanguineus* from aqueous solution: Equilibrium and kinetic studies. J Hazard Mater. 2009;161(1):189–95. doi: 10.1016/j.jhazmat.2008.03.104 18513859

[72] Bayramoǧlu G, Bektaş S, Arica MY. Biosorption of heavy metal ions on immobilized white-rot fungus *Trametes versicolor*. J Hazard Mater. 2003;101(3):285–300. doi: 10.1016/s0304-3894(03)00178-x 12935760

[73] Iqbal M, Edyvean RGJ. Biosorption of lead, copper and zinc ions on loofa sponge immobilized biomass of *Phanerochaete chrysosporium*. Miner Eng. 2004;17(2):217–23.

[74] Li X, Liao D, Xu X, Yang Q, Zeng G, Zheng W, et al. Kinetic studies for the biosorption of lead and copper ions by *Penicillium simplicissimum* immobilized within loofa sponge. 2008;159(2–3):610–5. doi: 10.1016/j.jhazmat.2008.02.068 18403109

[75] Tan WS, Ting ASY. Efficacy and reusability of alginate-immobilized live and heat-inactivated *Trichoderma asperellum* cells for Cu (II) removal from aqueous solution. Bioresour Technol. 2012 Nov;123:290–5. doi: 10.1016/j.biortech.2012.07.082 22940332

[76] Chen SH, Cheow YL, Ng SL, Ting ASY. Mechanisms for metal removal established via electron microscopy and spectroscopy: a case study on metal tolerant fungi *Penicillium simplicissimum*. J Hazard Mater. 2019 Jan;362:394–402. doi: 10.1016/j.jhazmat.2018.08.077 30248661

[77] Wan S, Ma Z, Xue Y, Ma M, Xu S, Qian L, et al. Sorption of lead (II), cadmium (II), and copper (II) ions from aqueous solutions using tea waste. Ind Eng Chem Res. 2014 Feb;53(9):3629–3635.

[78] Oh J-J, Kin JY, Kin YJ, Kin S, Kin G-H. Utilization of extracellular fungal melanin as an eco-friendly biosorbent for treatment of metal-contaminated effluents. Chemosphere. 2021 Jun;362:129884. doi: 10.1016/j.chemosphere.2021.129884 33582504

[79] Simonescu CM, Ferdeş M. Fungal Biomass for Cu(II) Uptake from Aqueous Systems. Pol. J. Environ. Stud. 2012;21(6):1831–9.

[80] Xu X, Zhang Z, Huang Q, Chen W. Biosorption Performance of Multimetal Resistant Fungus *Penicillium chrysogenum* XJ-1 for Removal of Cu^2+^ and Cr^6+^ from Aqueous Solutions. Geomicrobiol. J. 2018;35(1):40–9.

[81] Gadd GM. Biosorption: critical review of scientific rationale, environmental importance and significance for pollution treatment. J Chem Technol Biotechnol. 2009 May;84:13–28.

[82] Yuncu B, Sanin FD, Yetis U. An investigation of heavy metal biosorption in relation to C/N ratio of activated sludge. J Hazard Mater. 2006 Sep;137(2):990–997. doi: 10.1016/j.jhazmat.2006.03.020 16713077

[83] Ponou J, Wang LP, Dodbiba G, Okaya K, Fujita T, Mitsuhashi K, et al. Recovery of rare earth elements from aqueous solution obtained from Vietnamese clay minerals using dried and carbonized *parachlorella*. J Environ Chem Eng. 2014 Jun;2(2):1070–81.

