# Supplementary material for: Innovative method for encapsulating highly pigmented biomass from Aspergillus nidulans mutant for copper ions removal and recovery
Source: PLoS One. 2021 Nov 2;16(11):e0259315. doi: 10.1371/journal.pone.0259315 (PMC8562857; doi:10.1371/journal.pone.0259315)
Supplement: S1 Table — (DOCX) [file pone.0259315.s002.docx]

**S1 Table. The biosorption capacity of copper (q) from the pseudo-first and pseudo-second order models for encapsulated biosorbents (EB10 and EB30) and control as a function of the biosorption time.**

| Time (min) | EB30 | | | EB10 | | | Control^a^ |
| --- | --- | --- | --- | --- | --- | --- | --- |
|  | q_exp_ (mg g^-1^)^b^ | q_1_ (mg g^-1^)^c^ | q_2_ ( mg g^-1^)^d^ | q (mg g^-1^)^b^ | q_1_ ( mg g^-1^)^c^ | q_2_ ( mg g^-1^)^d^ | (mg L^-1^)^e^ |
| 0 | 0.00 | -0,36 | - | 0.00 | -11.71 | - | 89.24 |
| 20 | 3.52 ± 0.04 | 3.42 | 4.28 | 5.66 ± 1.05 | -1.83 | 5.05 | 87.65 |
| 60 | 9.28 ± 0.77 | 8.05 | 8.18 | 9.86 ± 1.97 | 10.24 | 11.20 | 88.31 |
| 120 | 10.67 ± 0.83 | 11.12 | 10.59 | 15.35 ± 0.74 | 18.19 | 16.10 | 87.76 |
| 180 | 11.73 ± 0.54 | 12.23 | 11.75 | 18.34 ± 1.23 | 21.06 | 18.85 | 87.43 |
| 240 | 12.73 ± 0.45 | 12.64 | 12.42 | 22.49 ± 2.33 | 22.10 | 20.61 | 87.39 |
| 300 | 12.97 ± 1.43 | - | 12.87 | 23.48 ± 2.42 | - | 21.83 | 86.35 |
| 360 | 12.92 ± 0.95 | - | 13.19 | 22.11 ± 1.26 | 22.62 | 22.73 | 89.20 |
| 420 | - | - | - | 22.69 ± 1.90 | 22.67 | 23.41 |  |

^a^ Control: cellulose membrane without biomass.

^b^ q_exp_: Experimental biosorption capacity (values are the means ± standard deviation of three independent experiments).

^c^ q_1_: Biosorption capacity from pseudo-first order model.

^d^ q_2_: Biosorption capacity from pseudo-second order model.

^e^ Copper concentration after biosorption.
