# Supplementary material for: Innovative method for encapsulating highly pigmented biomass from Aspergillus nidulans mutant for copper ions removal and recovery
Source: PLoS One. 2021 Nov 2;16(11):e0259315. doi: 10.1371/journal.pone.0259315 (PMC8562857; doi:10.1371/journal.pone.0259315)
Supplement: S2 Table — (DOCX) [file pone.0259315.s003.docx]

**S2 Table.  The biosorption capacity of copper (q) from Langmuir and Freundlich models for encapsulated biosorbents (EB10, EB10x, EB30 and EB30x) and free biomass at different copper concentrations at equilibrium.**

| [Cu] (mg L^-1^)^a^ | q_exp_ (mg g^-1^)^b^ | | q_Langmuir_  (mg g^-1^) | | q_Freundlich_ (mg g^-1^) | |
| --- | --- | --- | --- | --- | --- | --- |
|  | EB10 | EB10x | EB10 | EB10x | EB10 | EB10x |
| 138.77 | 33.15 ± 1.40 | 19.13 ± 1.41 | 31.90 | 19.11 | 33.42 | 19.97 |
| 273.23 | 56.58 ± 3.39 | 28.98 ± 3.52 | 57.48 | 29.50 | 56.29 | 28.18 |
| 432.58 | 80.86 ± 3.15 | 37.17 ± 3.12 | 82.69 | 37.19 | 80.17 | 35.59 |
| 550.21 | 96.70 ± 2.60 | 41.13 ± 2.96 | 98.53 | 41.11 | 96.47 | 40.22 |
| 689.27 | 115.85 ± 3.02 | 46.23 ± 3.12 | 114.85 | 44.61 | 114.74 | 45.10 |
| 862.33 | 133.95 ± 2.09 | 46.84 ± 2.14 | 132.25 | 47.84 | 136.32 | 50.54 |
| [Cu] (mg L^-1^)^a^ | EB30 | EB30x | EB30 | EB30x | EB30 | EB30x |
| 28.85 | 15.81 ± 1.00 | 14.85 ± 1.04 | - | - | - | - |
| 112.11 | 22.35 ± 3.12 | 18.61 ± 3.07 | 19.28 | 16.71 | 21.86 | 18.63 |
| 222.75 | 31.62 ± 4.49 | 24.19 ± 4.57 | 32.91 | 25.53 | 32.58 | 24.81 |
| 339.89 | 40.63 ± 4.81 | 29.30 ± 4.24 | 43.70 | 31.29 | 41.65 | 29.59 |
| 479.79 | 50.85 ± 3.20 | 34.86 ± 3.46 | 53.41 | 35.77 | 50.90 | 34.17 |
| 636.78 | 63.33 ± 2.29 | 42.10 ± 2.54 | 61.61 | 39.12 | 60.00 | 38.45 |
| 797.82 | 68.63 ± 1.17 | 42.04 ± 0.55 | 68.06 | 41.53 | 68.41 | 42.25 |
| 935.50 | 73.27 ± 1.23 | 42.09 ± 1.92 | 72.47 | 43.06 | 75.04 | 45.15 |
| [Cu] (mg L^-1^)^a^ | Free fungal biomass | - | | | | |
| 24.19 | 27.68 ± 0.31 | - | - | - | - | - |
| 102.40 | 41.82 ± 3.66 | - | - | - | - | - |
| 220.41 | 59.13 ± 4.35 | - | - | - | - | - |
| 315.60 | 78.52 ± 5.63 | - | - | - | - | - |
| 489.49 | 91.97 ± 7.36 | - | - | - | - | - |
| 621.25 | 91.85 ± 7.11 | - | - | - | - | - |

^a^ Copper concentration at equilibrium.

^b^q_exp_: Experimental biosorption capacity (values are the means ± standard deviation of three independent experiments).
