# Supplementary material for: Innovative method for encapsulating highly pigmented biomass from Aspergillus nidulans mutant for copper ions removal and recovery
Source: PLoS One. 2021 Nov 2;16(11):e0259315. doi: 10.1371/journal.pone.0259315 (PMC8562857; doi:10.1371/journal.pone.0259315)
Supplement: S3 Table — (DOCX) [file pone.0259315.s004.docx]

**S3 Table. Desorption capacity (q_des_) and recovery efficiency of copper by the encapsulated biosorbent (EB30) as a function of desorption time.**

| Time (min.) | q_des_ (mg g^-1^) | Recovery efficiency (%) |
| --- | --- | --- |
| 30 | 17.99 | 32.57 |
| 60 | 25.56 | 46.17 |
| 120 | 33.83 | 61.19 |
| 180 | 36.35 | 65.83 |
| 240 | 37.81 | 68.48 |
| 300 | 37.62 | 68.17 |
| 360 | 37.71 | 68.28 |
