# Supplementary material for: Innovative method for encapsulating highly pigmented biomass from Aspergillus nidulans mutant for copper ions removal and recovery
Source: PLoS One. 2021 Nov 2;16(11):e0259315. doi: 10.1371/journal.pone.0259315 (PMC8562857; doi:10.1371/journal.pone.0259315)
Supplement: S4 Table — (DOCX) [file pone.0259315.s005.docx]

**S4 Table. Biosorption (q) and desorption (q_des_) capacity and recovery efficiency of copper by the encapsulated biosorbent (EB30) as a function of successive cycles.**

| Cycles | q (mg g^-1^)^a^ | q_des_ (mg g^-1^)^a^ | Recovery efficiency (%)^a^ |
| --- | --- | --- | --- |
| I | 52.93 ± 2.81 | 36.57 ± 1.54 | 69.32 ± 6.63 |
| II | 52.00 ± 3.67 | 33.96 ± 0.74 | 65.59 ± 6.14 |
| III | 52.87 ± 3.33 | 33.63 ± 1.08 | 63.69 ± 1.94 |
| IV | 58.40 ± 9.23 | 35.97 ± 0.56 | 62.75 ± 11.05 |
| V | 49.60 ± 2.77 | 35.43 ± 0.74 | 71.59 ± 4.27 |

^a^Values are the means ± standard deviation of three independent experiments.
